# Multibacillary Mid-Borderline Leprosy with Type 1 Lepra Reaction and Concurrent Latent Tuberculosis

**DOI:** 10.4269/ajtmh.21-0624

**Published:** 2021-10-25

**Authors:** Rusheng Chew, Marion L. Woods

**Affiliations:** Infectious Diseases Unit, Royal Brisbane and Women’s Hospital, Herston, Queensland, Australia; and Faculty of Medicine, University of Queensland, Herston, Queensland, Australia

**Figure 1.**
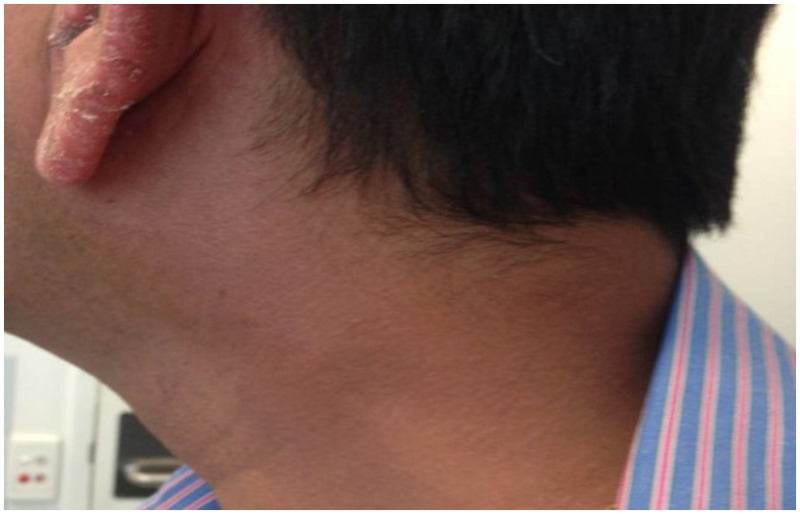
Enlarged left greater auricular nerve. This was initially diagnosed elsewhere as thrombophlebitis. This figure appears in color at www.ajtmh.org.

**Figure 2.**
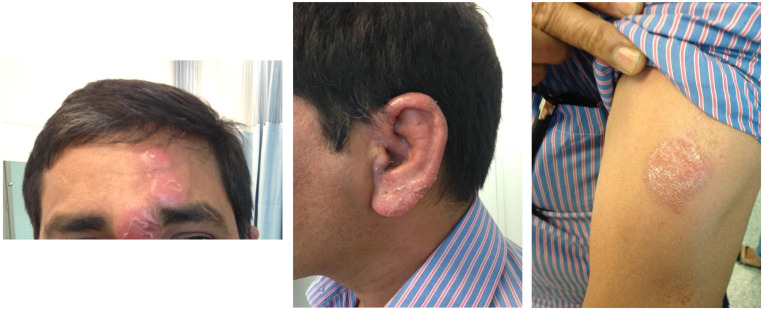
Type 1 lepra reaction lesions on the (left) forehead, (middle) left pinna, and (right) left upper arm before treatment with prednisone. This figure appears in color at www.ajtmh.org.

A 52-year-old male abattoir worker originally from India presented with a 9-month history of a tender, paresthetic, cord-like, left-side neck lump diagnosed elsewhere as thrombophlebitis but had failed to improve after several months of conservative treatment (Figure 1). The patient was otherwise asymptomatic, but physical examination revealed six scaly, hypopigmented non-pruritic and non-hypo-esthetic plaques on the forehead, left pinna, left upper arm, and right thigh in an asymmetrical distribution. Two months later, these lesions became spontaneously painful, erythematous, and edematous (Figure 2).

Computed tomography of the neck to pelvis showed only mild lymphadenopathy in the right supraclavicular and deep cervical chains. The neck lump was identified as an inflamed left greater auricular nerve. Interferon-γ release assay for tuberculosis was positive, but active tuberculosis was excluded by clinical and radiological assessment.

**Figure 3.**
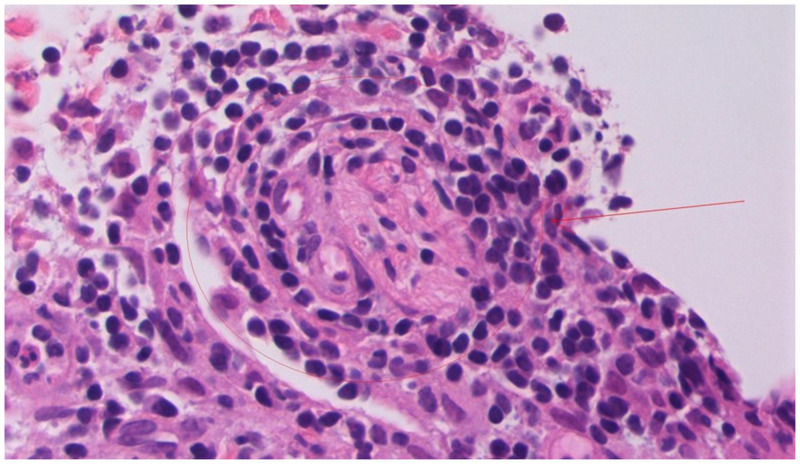
Hematoxylin and eosin-stained punch biopsy of type 1 lepra reaction lesion showing granulomatous dermatitis with perivascular and peri-adnexal granuloma around nerve fibers (arrow). Polymerase chain reaction testing for *Mycobacterium leprae* was positive. This figure appears in color at www.ajtmh.org.

Histopathological examination of lesion punch biopsies showed granulomatous dermatitis, with perivascular and peri-adnexal granulomas around nerve fibers (Figure 3). Wade-Fite stain for acid-fast bacilli was negative, but polymerase chain reaction for *Mycobacterium leprae* was positive, confirming the diagnosis of leprosy.

The diagnosis was further defined as multibacillary mid-borderline leprosy based on clinical appearance, number and distribution of skin lesions, and histopathological findings,^1^^,^^2^ with concurrent latent tuberculosis. The acute inflammatory skin lesion changes were classic for type 1 lepra reaction (Table 1). Clofazimine, 50 mg/d and 300 mg/month; rifampicin, 600 mg/month; and dapsone 100 mg/d for 12 months were commenced per WHO guidelines,^1^ with a 9-month course of isoniazid, 300 mg/d, for latent tuberculosis. A weaning course of prednisone starting at 20 mg/d for the type 1 lepra reaction was commenced after treatment of latent tuberculosis had begun.

**Table 1:** Definitions of terms pertaining to leprosy used in the main text

Term	Definition
Multibacillary leprosy	All cases with positive slit-skin smears, or more than five skin lesions where slit-skin smears are not available. If in doubt, treat as multibacillary leprosy to avoid under-treatment.^6^
Borderline leprosy	An immunologically unstable form of leprosy with features between the two poles of tuberculoid (localized) and lepromatous (disseminated) disease. Borderline leprosy encompasses borderline tuberculoid, borderline borderline, and borderline lepromatous disease.
Type 1 lepra reaction	A delayed hypersensitivity reaction characterized by swelling and redness of skin lesions.^6^ Also known as “reversal reaction,” it may occur in any form of leprosy, but borderline leprosy carries the highest risk as a result of its immunological instability.

**Figure 4.**
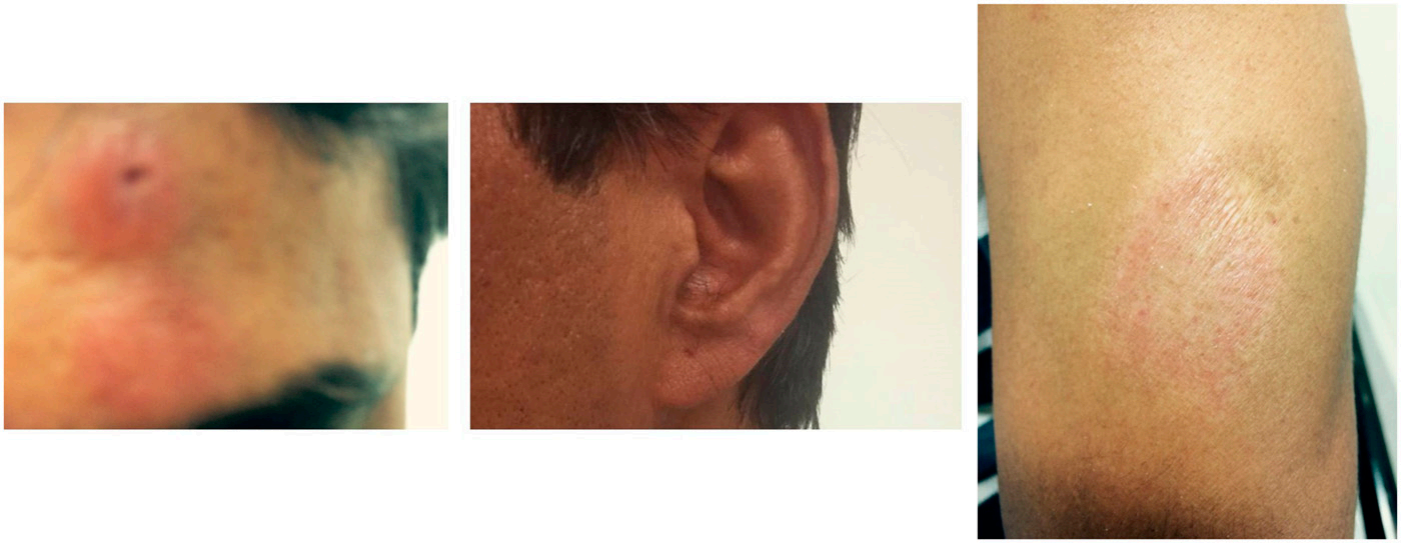
Type 1 lepra reaction lesions on the (left) forehead, (middle) left pinna, and (right) left upper arm showing marked improvement in appearance after 11 months of treatment with prednisone and combination anti-leprotic therapy. This figure appears in color at www.ajtmh.org.

The patient completed therapy for latent tuberculosis successfully, and at 11 months post-commencement of steroid and anti-leprotic therapy, the skin lesions had improved markedly (Figure 4).

Clinicians should be aware that the greater auricular nerve runs parallel to the external jugular vein and may be mistaken for it.^3^ It may be involved in leprosy, but it is purely sensory,^3^^,^^4^ which may delay presentation as a result of lack of symptoms apart from pain or paresthesia. These pitfalls likely contributed to the initial misdiagnosis of thrombophlebitis. Furthermore, leprotic skin lesions may not always be hypo-esthetic.

The prevalence of type 1 lepra reactions in patients with borderline leprosy has been estimated to be as high as 35%,^5^ and early anti-inflammatory treatment prevents further nerve damage and loss of function. It has been proposed that additional mycobacterial antigenic triggers, for example from tuberculosis, may increase the risk of type 1 lepra reaction.^5^ As in our patient, a considerable proportion of patients with leprosy come from countries with a high prevalence of tuberculosis; thus, clinicians should have a low threshold to exclude concurrent tuberculosis.
